# LAG-3 potentiates the survival of *Mycobacterium tuberculosis* in host phagocytes by modulating mitochondrial signaling in an in-vitro granuloma model

**DOI:** 10.1371/journal.pone.0180413

**Published:** 2017-09-07

**Authors:** Bonnie L. Phillips, Uma S. Gautam, Allison N. Bucsan, Taylor W. Foreman, Nadia A. Golden, Tianhua Niu, Deepak Kaushal, Smriti Mehra

**Affiliations:** 1 Tulane National Primate Research Center, Covington, Louisiana, United States of America; 2 Department of Microbiology & Immunology, Tulane University School of Medicine, New Orleans, Louisiana, United States of America; 3 Department of Biostatistics and Bioinformatics, Tulane University School of Public Health, New Orleans, Louisiana, United States of America; 4 Department of Pathobiological Sciences, Louisiana State University School of Veterinary Medicine, Baton Rouge, Louisiana, United States of America; Jawaharlal Nehru University, INDIA

## Abstract

CD4^+^ T-cell mediated Th1 immune responses are critical for immunity to TB. The immunomodulatory protein, lymphocyte activation gene-3 (LAG-3) decreases Th1-type immune responses in T-cells. LAG-3 expression is significantly induced in the lungs of macaques with active TB and correlates with increased bacterial burden. Overproduction of LAG-3 can greatly diminish responses and could lead to uncontrolled *Mtb* replication. To assess the effect of LAG-3 on the progression of *Mtb* infection, we developed a co-culture system wherein blood-derived macrophages are infected with *Mtb* and supplemented with macaque blood or lung derived CD4^+^ T-cells. Silencing LAG-3 signaling in macaque lung CD4^+^ T-cells enhanced killing of *Mtb* in co-cultures, accompanied by reduced mitochondrial electron transport and increased IFN-γ expression. Thus, LAG-3 may modulate adaptive immunity to *Mtb* infection by interfering with the mitochondrial apoptosis pathway. Better understanding this pathway could allow us to circumvent immune features that promote disease.

## Introduction

*Mtb*, the causative agent of TB results in approximately 1.4 million deaths annually [1, 2]. Additionally, 9 million individuals are newly infected with *Mtb* each year [3]. The interaction between the phagocytes and T-cells within the lung granuloma are key for the control of *Mtb*. Modulation of the Th1 immune response, which can potentially sterilize infection, is required for control of tissue damaging immunopathology, but may provide the bacillus with a niche to persist [4]. Immunosuppression can modulate T-cell function through a decreased ability to recognize antigen, activate, proliferate, produce cytokines or eventually, increased exhaustion [5–7], potentially resulting in the loss of containment of *Mtb* [8, 9].

The lung granuloma is crucial in the determination of whether *Mtb* infection results in control or progression of TB [10]. The classical *Mtb* induced lung granuloma in humans is a stratified, well-organized structure. It consists of a central region containing *Mtb*-infected monocyte-derived cells [11] and is surrounded by uninfected monocyte-derived cells, including alveolar macrophages, dendritic cells, and epitheleoid macrophages. These are in turn encircled by lymphocytic cells, such as T and B cells [12]. Both phagocytic cells and T-cells are key for the control of *Mtb*. Phagocytic cells; especially macrophages are part of the first line of defense against the pathogen. After uptake of the bacillus, these cells can inhibit mycobacterial replication and even neutralize the pathogen itself through a plethora of mechanisms including phagosomal maturation and phagolysosomal fusion, with bactericidal activity due to acidification, and reactive oxygen and nitrogen intermediates [13–15]. T cells are also indispensable in their action towards the containment of *Mtb*, where T-cells primarily function through the production of Th1 cytokines [16–18]. The release of IFN-γ, TNF-α, and IL-2 allow for activation of macrophages, immune cell recruitment, and greater T cell proliferation [19, 20].

While too great an inflammatory response may lead to immunopathogenesis, resulting in the loss of integrity in the granuloma structure [21, 22], an overabundance of immunomodulatory molecules can also cause immunosuppression, e.g. with IL-10 overexpression [23]. In either case, the end result is a loss of control of bacterial replication. An increased presence of these immunosuppressive molecules can cause decreased T cell function through a decreased ability to recognize antigen, become activated, proliferate, produce cytokines or eventually though exhaustion [5–7]. Any of the above could lead to the loss of containment of *Mtb*, and evasion of the pathogen from the immune response [8, 9].

LAG-3 acts as a negative co-stimulatory receptor (checkpoint inhibitor) that inhibits immune response by competitively inhibiting the CD4-MHC-II antigen presentation interaction [24]. LAG-3 dampens the Th1 immune response through the activation and resulting proliferation of Tregs, T cell dysregulation, as well has the inhibition of monocyte differentiation; both of which have a deleterious downstream effect on Th1 effector T-cell activation, proliferation and function [7, 24–26]. Blocking of LAG-3 signaling resulted in increased antigen presentation creating an elevated Th1 response with increased production of IFN-γ [27]. LAG-3 expressing T-cells are functionally exhausted and correlate with HIV disease in macaques [28]. As a checkpoint inhibitor, LAG-3 depletion is currently in clinical trials for cancer immunotherapy [29, 30]. LAG-3 may be a more appropriate checkpoint inhibitor target than PD-1 since interference with the latter only results in the activation of effector T-cells, while antagonizing the former can additionally inhibit the suppressive action of Tregs.

LAG-3 is significantly induced (~100 fold) in human like macaque lungs during active TB [31, 32] and is specifically localized to groups of T-cells, including Tregs, as well as NK cells [32]. LAG-3 expression in macaque lungs correlates with higher *Mtb* burden [31, 32]. Furthermore, majority of LAG-3 expression occurred on CD4^+^ T-cells in the lung granuloma, in macaques with active TB and to a lower extent, in animals where LTBI was reactivated due to co-infection with SIV. Further, these cells also co-expressed IL-10. LAG-3 was not however, expressed in lungs of animals with LTBI, or animals infected with SIV or pulmonary bacterial pathogens other than *Mtb*. Together, these data suggest a potential role for this known modulator of Th1 responses in TB [7, 24]. Thus, a strong rationale exists for studying the role played by LAG-3 in negatively regulating immune responses in TB.

We sought to understand the role of LAG-3 in modulating host responses to TB with a simplistic co-culture model consisting of *Mtb*-infected differentiated macaque macrophages and CD4^+^ T cells—the two major cellular populations within a granuloma. CD4^+^ cells were derived from either the blood or the lungs of *Mtb*-infected NHPs. siRNA was used to silence the expression of LAG-3 within a subset of CD4^+^ cells before co-culturing (relative to controls with scrambled, nonspecific siRNA). *In-vitro* models of *Mtb*-infected macrophages have been extensively employed [33–35]. The aim was to understand if their interaction with CD4^+^ cells could mimic *in-vivo* interactions. Further, we sought to specifically understand if blockading LAG-3 signaling would have a perceivable impact on the function of CD4^+^ cells (e.g. greater activation), resulting in the control of *Mtb*. To test this hypothesis, we measured *Mtb* burdens in such co-cultures over the course of time (0–96 hrs) and assessed T cell phenotype, cytokine production and transcriptomics at specific time-points where samples could be banked.

## Materials and methods

The Tulane National Primate Research Center Institutional Animal Care and Use Committee (IACUC) and the Tulane Institutional Biosafety Committee (IBC) approved all procedures.

### Co-cultures

PBMCs were isolated from EDTA treated blood of infected rhesus macaques and plated into 12-well Poly-L-Lysine coated plates in antibiotic containing complete RPMI for 4h to allow for adherence and then differentiated for 120h before *Mtb* infection [36]. T-cells were collected from both the blood and dematricized [37] lungs of animals with active TB [32, 38, 39]. Both blood and lung tissue was collected from macaques on an approved and previously completed study [40] and banked for future use. CD4^+^ T-cells were isolated via density gradient centrifugation using Histopaque-1077 using negative selection with a MACS NHP CD4^+^ T-cell Isolation Kit (Miltenyi). Mononuclear cells were passed through MS columns affixed to the OctoMACS^™^ Separator Magnet and the unlabeled CD4^+^ T-cell population collected and cryopreserved. The differentiated macrophages were infected with *Mtb* at an MOI of 5:1 for 4h [33, 34, 41], which was deemed time-point 0. CD4^+^ T-cells were then supplemented to the culture at a 1:1 ratio to macrophages (~5x10^5^ CD4^+^ T-cells) (S1 Fig). Samples were collected at 0, 4, 24, 48, 72, and 96h post-infection for CFU as described earlier [33, 34, 41].

### LAG-3 siRNA transfection in CD4^+^ T-cells

siRNA transfection has previously been described [34, 42]. siRNAs for LAG-3, Cyclophilin B as positive control, and non-specific negative control (S2 Fig) were combined with the transfection reagent (Dharmacon) for 20 minutes and added to CD4^+^s, which were then incubated for 24h before being added to *Mtb*-infected macrophage culture. *Mtb* CFU assays in cultures were performed as described earlier [33, 34, 41].

### Flow cytometry, confocal microscopy and cytokine assays

Flow cytometry was performed on co-cultured T-cells as previously described [31, 32, 40, 43–45] (S1 Table). Confocal microscopy was performed on fixed adherent differentiated macrophages and co-cultures [32–34, 38] and cytokine assays on supernatant, as described earlier [32–34, 42]. *Mtb*-specificity of blood or lung-derived T-cells was identified by stimulating with either a positive control, or a negative control or *Mtb* CW (BEI Resources) as previously described [40, 46].

### RNA extraction, quantitative RT-PCR and transcriptomics

RNA extraction and cDNA synthesis were performed as described earlier [32–34, 41]. RT-PCR was performed in duplicate and data analyzed as described earlier [32]. RNA was amplified and used for microarrays [31, 38, 40, 44, 47–49]. RNA from uninfected macrophages was used as baseline. For statistical analysis of pathways and categories, we used DAVID (The Database for Annotation, Visualization and Integrated Discovery) v6.7 (https://david.ncifrcf.gov) [40, 44]. Genes with at least a two-fold induction were uploaded to DAVID to identify statistically significant accumulation of genes across Gene Ontology. Gene expression data was uploaded to Gene Expression Omnibus (GEO) and can be retrieved using the accession number (GPL10183).

### Statistics

For most analyses, we first determined if the data were normally distributed or not. Since in almost all instances, data did not significantly depart from normality, we used a non-parametric Mann-Whitney U test to assess statistical significance of the results. For transcriptomics analyses, we identified terms with false-discovery rate (FDR) corrected p-values of 0.05 or less as significantly accumulated. *P*-values were transformed to negative log_10_ values to provide visual assessment of the magnitude of significant shift.

## Results

### Establishment of the *Mtb*-infected macrophage-T-cell co-culture model

**Fig 1:**
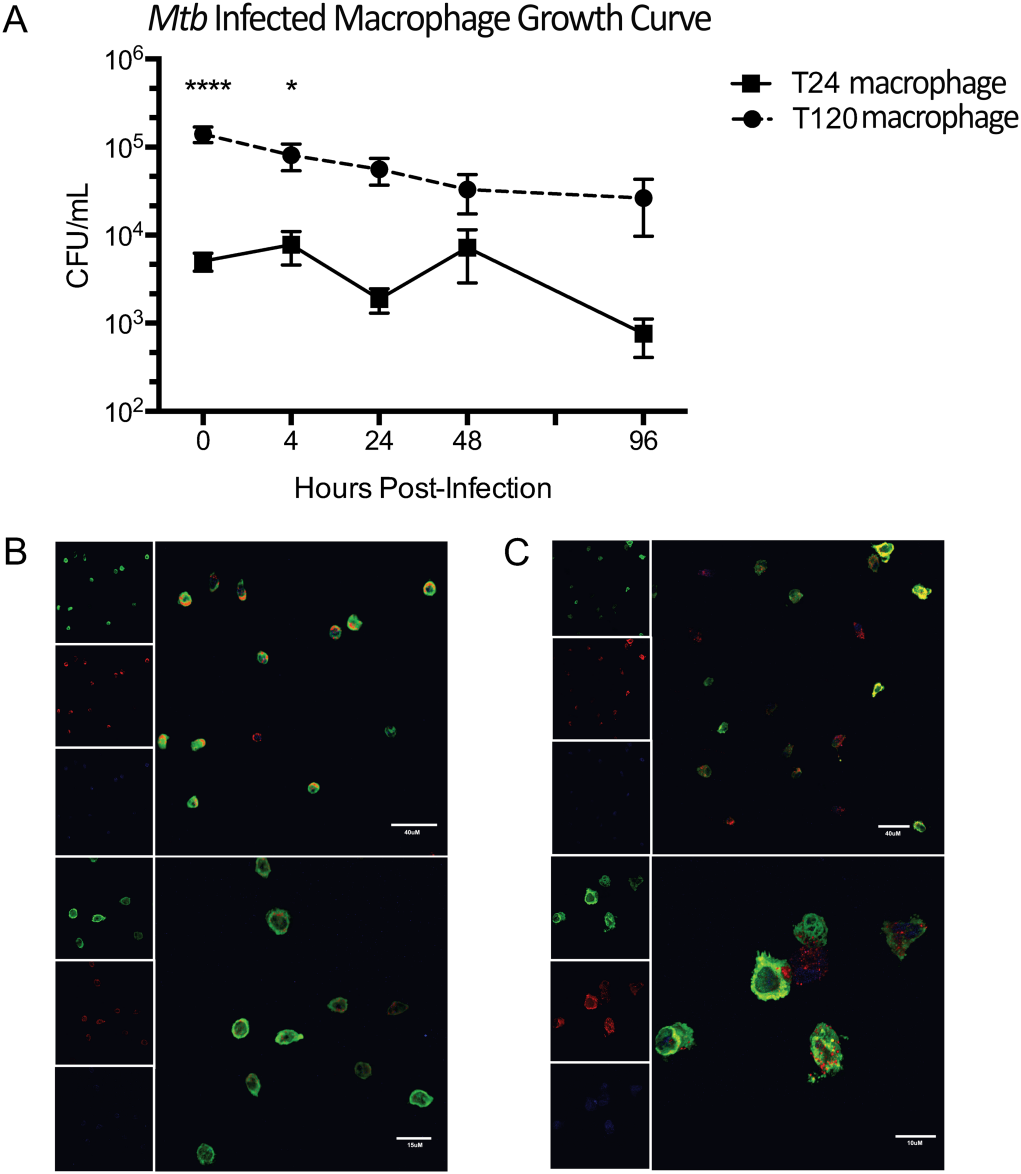
Comparison of *Mtb*-infected T24 monocytes vs. T120 differentiated macrophages. (A) Log_10_-bacterial burden at 0, 4, 24, 48 72, and 96h post-infection. T24 monocytes are indicated by red squares and blue circles indicate T120 differentiated macrophages infected with *Mtb*. Cellular morphology and bacterial uptake at time-point 0 in *Mtb*-infected phagocytes that were allowed to differentiate for (B) 24h and (C) 120h before infection. Cells were stained for MAC387 (green), *Mtb* (red), and To-PRO-3 (blue). Upper panels show medium magnification (40x), and lower panels indicate high magnification (63x). Data are means ± SEM from 4 or 6 independent experiments. In Prism v6.0, a Two-Way ANOVA with Sidak’s multiple comparisons test was performed where ****, P<0.0001; *, P<0.05.

We first compared *Mtb* infection in NHP blood-derived monocytes differentiated for 24h (T24 monocytes) versus macrophages differentiated for 120h (T120 differentiated macrophages) (S1 Fig). The latter were more efficient at initial bacterial uptake when compared to T24 monocytes (Fig 1A), with an initial bacterial burden of 1.7x10^5^ CFU/mL, ~25 times greater than in the T24 monocyte group (5.0x10^3^ CFU/mL) (*P*>0.0001). Since the data were not significantly departed from normality, we used a non-parametric Mann-Whitney U test to assess the statistical significance of these results. The differences between the T24 monocyte and the T120 macrophage groups were significant at 0 (*P*<0.01), 4 (*P*<0.01) and 24 (*P*<0.05) hrs. The differences at 0 and 4 hr were also statistically significant after the application of Bonferroni correction.

The cellular morphology of T24 monocytes was typical of undifferentiated monocytes (Fig 1B), with all cells appearing fairly homogenous and maintaining a round shape. In contrast, the cells differentiated for 120h had heterogeneous morphology with signs of differentiation, including macrophage-like characteristics such as: elongated cell bodies, and diffused and amoeboid morphology (Fig 1C). Also, the readily visible presence of *Mtb* within the phagocytes reinforced the bacterial burden data at time-point 0 as assessed by bacterial burden (Fig 1B and 1C). Thus, our data suggested that the T120 differentiated macrophages were ideally suited for use in a co-culture model involving *Mtb* infection. Such a model offers a simplistic overview of the interaction between the two major cell types within the granuloma, *in vitro*. Direct recognition of infected mononuclear cells by T-cells is essential for the control of *Mtb* [50]. Such models can facilitate mechanistic studies needed to ascertain the role of LAG-3 in modulating anti-*Mtb* immune responses [24].

**Fig 2:**
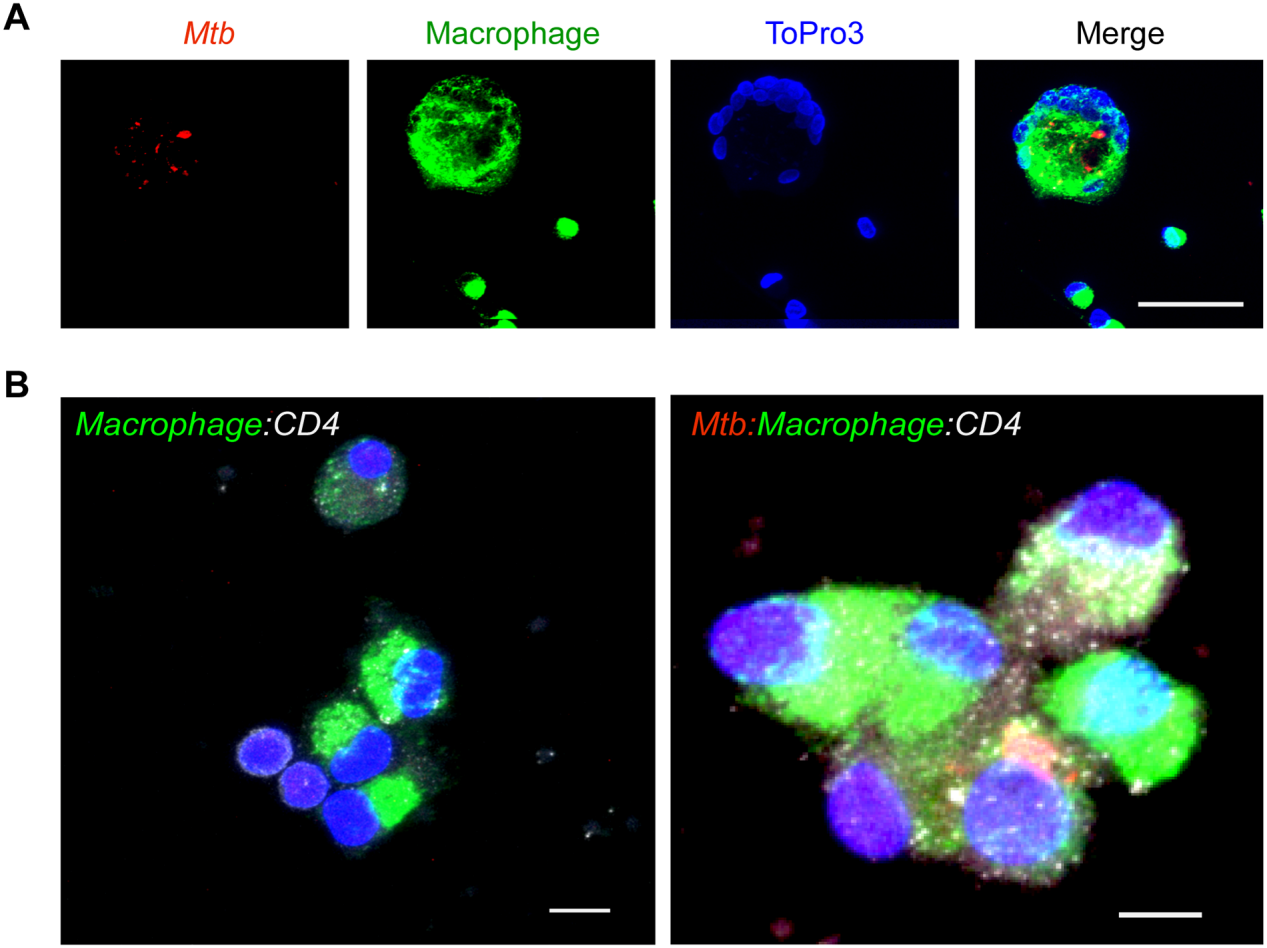
Interaction between macaque macrophages and CD4^+^ T-cells during co-culture is shown using multilabel confocal microscopy. Immunostaining of cells positive for *Mtb* (red), macrophages (green), nuclei (blue) and a merge images (far right) (A). A representative image of macrophage (green):CD4^+^ T-cell (white) co-culture (lower left panel) and an infected macrophage with *Mtb* and associated with T-cell (lower right panel) (B); scale bars- 100 μm (A), 20 μm and 40 μm (B).

We also assessed the interaction between co-cultured macrophages and T-cells (Fig 2). Co-cultures were stained for macrophages using a combination of CD68 and CD163 (in green), *Mtb* using a specific antibody (red) and for non-macrophage cells (T-cells) using nucleolar marker ToPro3 (blue) (Fig 2A) [31–34, 38, 39, 47, 49, 51–53]. T-cells accumulated in the outer peripheral region of these co-cultures (Fig 2A). We next stained for macrophages (CD68^+^CD163^+^, green), ToPro3 (blue), *Mtb* (red) and CD4s using an anti-CD4 antibody (white) (Fig 2B) and were able to demonstrate the interaction between macrophages that engulfed *Mtb* and CD4^+^ T cells (Fig 2B). These results validate our co-culture model as a macaque version of the various in-vitro granuloma models that have recently been developed [54, 55]. We further investigated whether some of the T cells derived from the whole blood (via PBMCs) or lungs of macaques were *Mtb*-specific. One million PBMCs or lung cells each were stimulated as described earlier with either SEB as a positive control, or *Mtb* cell wall fraction (CW; BEI resources) or using a media-only negative control. We found that between 0.25–0.5% of all cells thus obtained (i.e. between 25,000–50,000 cells) in each sample were able to induce the production of IFN-γ in response to *Mtb* CW over and above negative control. While IFN-γ is not the correlate of antigen-specific cytokine production, these results strongly suggest that *Mtb*-specific T-cells were present in our co-cultures.

### Silencing LAG-3 in the co-culture system

LAG-3 expression was silenced in CD4^+^ T-cells alone using a specific siRNA at time-point 0 (S2 Fig) [24, 32]. Of the four siRNAs originally designed to silence LAG-3 expression, one resulted in significant (~85%) down-regulation of the intended target 24h post-transfection, (S2 Fig). A scrambled nonspecific siRNA served as a control [32].

### The effect of LAG-3 silencing on macrophage bacterial burden in co-cultures

**Fig 3:**
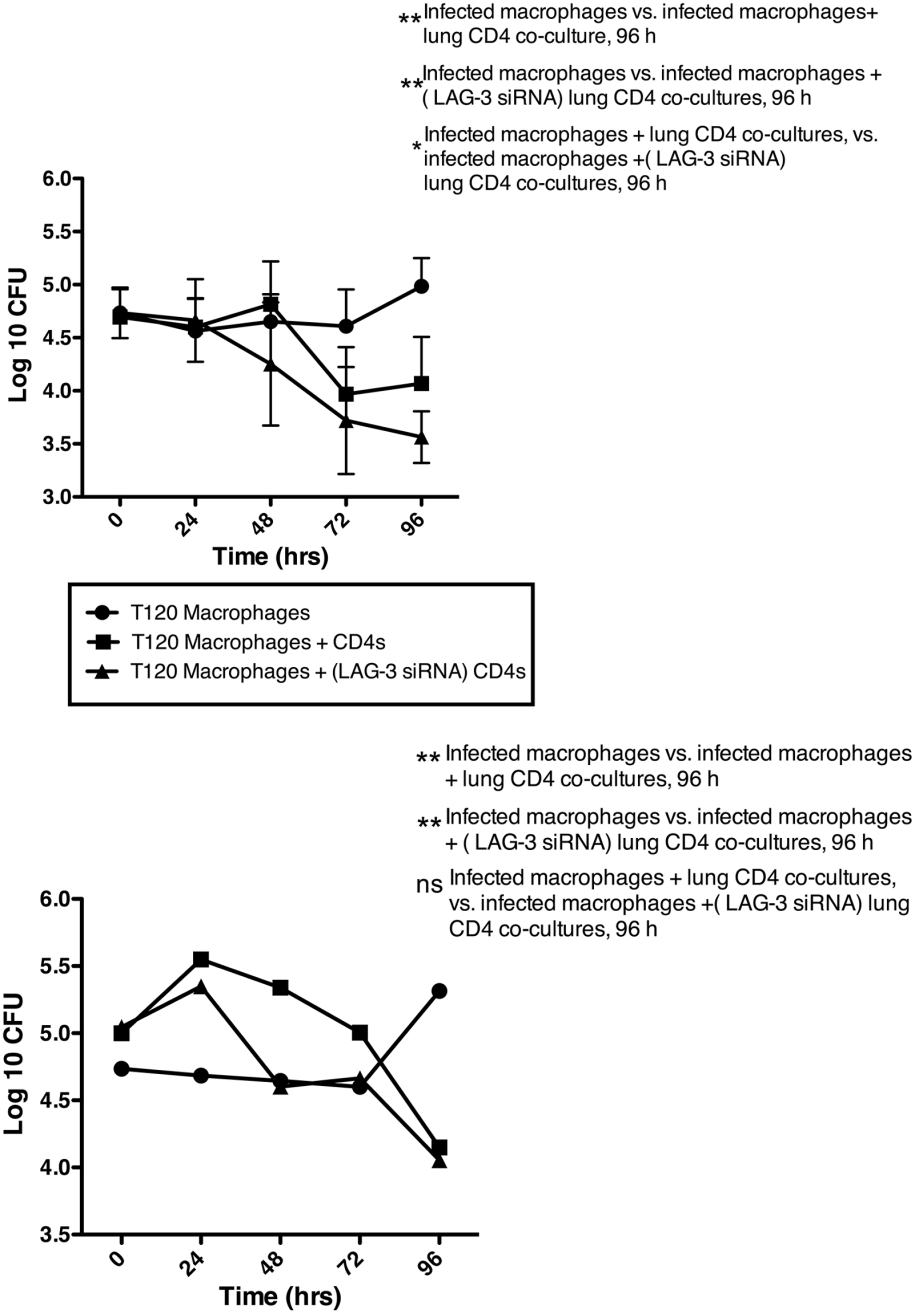
The effect of LAG-3 silencing on CD4^+^ T-cell killing of *Mtb* in a differentiated macrophage culture supplemented with CD4^+^ T-cells. Log_10_-bacterial burden at 0, 24, 48 72, and 96h post-infection in differentiated *Mtb*-infected macrophage cultures as well as co-cultures supplemented with CD4^+^ T-cells from blood (A) or lung (B) of *Mtb*-infected animals. The CD4^+^ T-cells were either untreated, or were silenced for LAG-3 with siRNA. The macrophage only cultures are indicated by solid circles, macrophage cultures supplemented with CD4^+^ T-cells are represented by solid squares, and macrophage cultures supplemented with LAG-3 silenced CD4^+^ T-cells are represented by solid triangles. Statistical significance was determined using a two-way ANOVA in Prism 6, with matched values stacked into sub columns, and using multiple comparisons to compare effects within each row (time) by comparing each experimental condition (macrophage, co-culture or LAG-3 RNAi co-culture) with every other condition at that time-point, with the Tukey multiple comparison test; the mean and SEM are represented by horizontal bars. *p < 0.05, **p < 0.01.

A dramatic decrease occurred in the macrophage bacterial burden upon co-culture with CD4^+^ T-cells, and this effect was observed for both blood (Fig 3A) and lung (Fig 3B) derived cells. While the bacterial load in macrophages alone remained constant for the first few days and then gradually increased, the load in T-cell co-cultures reduced progressively, with the lowest bacterial burden being found at the 96h time-point. Therefore, we were able to recapitulate the *in-vivo* interaction of infected macrophages and T-cells in the co-cultures. In several animal models, *Mtb* has been shown to replicate logarithmically for the first few weeks after experimental infection, and the onset of the adaptive response is able to significantly check this bacillary replication.

Moreover, silencing of LAG-3 expression reduced bacterial load over and above the effect of adding CD4^+^s to infected macrophages, with both the kinetics of bacterial killing and the magnitude of reduction in *Mtb* levels being greater when LAG-3 expression was silenced in CD4^+^s prior to co-culture. This effect was more pronounced in T-cells derived from the blood rather than the lungs of infected macaques. Hence, using two-factor repeated-measures ANOVA across time, the lower *Mtb* load in co-cultured macrophages as well as macrophages co-cultured with LAG-3 silenced blood-derived T-cells, was marginally significant (*P* = 0.0574892) relative to infected macrophages (*P*<0.01) at 96h (Fig 3A). While a comparable trend was observed in co-cultures set up with lung-derived T cells, the results were not statistically significant across time (Fig 3B). This result is not surprising given that LAG-3 expressions following *Mtb* infection are much higher in the lung and the effect of RNAi on LAG-3 transcripts were less pronounced in lung T cells. The decrease in bacterial burden over time was also greater for co-cultures with LAG-3 expression silenced. Thus, in these co-cultures, the bacterial load decrease was first observed at 48h when bacterial levels in regular co-cultures were comparable to the control macrophages. On the contrary, while the bacillary burden in co-cultures from lung derived T-cells was lower at 96h relative to the control group (*P*<0.05 both for regular and LAG-3 silenced co-cultures), there was no statistically significant difference between the burdens in the two types of co-cultures (*P*>0.05) (Fig 3B). We have earlier reported that the pro-inflammatory cytokine expression profile of active TB granulomas is ameliorated over time in macaques [31, 38], an observation supported by metabolic-imaging of lungs in this model [56]. Furthermore, very low frequencies of T-cells derived from the lungs of *Mtb*-infected macaques express protection-associated cytokines [57]. Taken together with these prior reports, the co-culture results suggest that the lung environment is anti-inflammatory during the chronic stages of *Mtb* infection, further underscoring the importance of LAG-3 and other checkpoint inhibitors, in this process.

### LAG-3 silencing in CD4^+^ T-cells and its role on expression of immunomodulatory proteins

**Fig 4:**
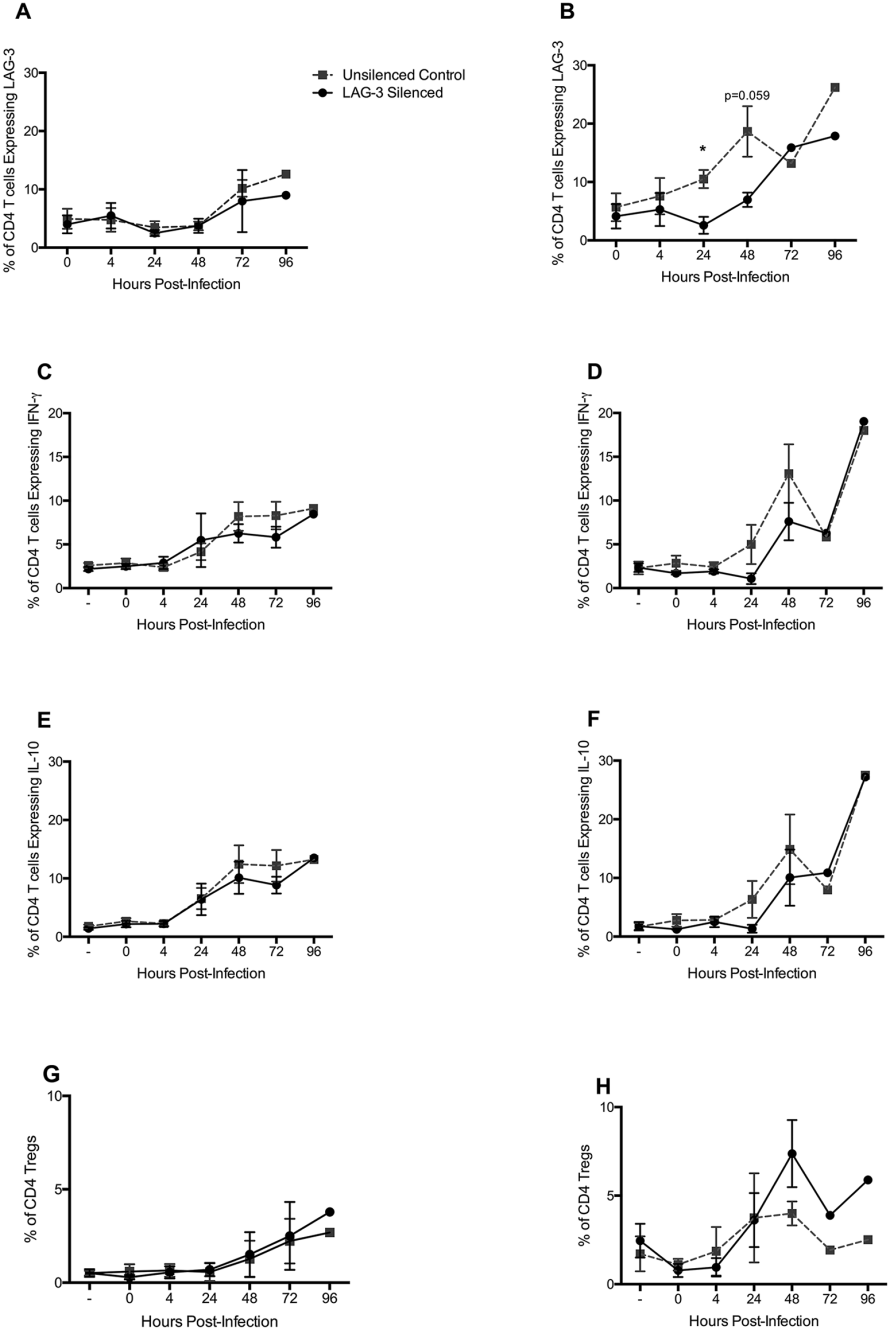
LAG-3 silencing and its effect on CD4^+^ T cells within the *Mtb*-infected co-culture. This data illustrates the mean frequency of CD4^+^ T cells positive for LAG-3 (**A**, **B**), IFN-γ (**C**, **D**), IL-10 (**E**, **F**), and Treg frequency (**G**,**H**) over the course of 96h in the *Mtb*-infected macrophage co-culture. In all images, gray squares indicate the *Mtb*-infected co-culture, where CD4^+^ T cell were untreated, and the black circles represent the *Mtb*-infected co-culture, where CD4^+^ T cell were silenced for LAG-3 before being added to the culture. In **A**, **C**, **E**, and **G** the CD4^+^ T cells used for co-culture were derived from blood of *Mtb*-infected rhesus macaques, whereas in **B**, **D**, **F**, and **H** the CD4^+^ T cells were isolated from lung of *Mtb*-infected rhesus macaques. Multiple t-tests corrected for multiple comparisons using the Holm-Sidak method were utilized to determine significance between time points. Horizontal bars represent the SEM. *P < 0.05.

Flow results were analyzed as shown (S3 Fig). In comparison to untreated blood-derived CD4^+^ T-cells, the frequency of LAG-3 positive *CD4* T-cells in LAG-3 silenced blood-derived T cells at times 72h and 96h post-infection, was essentially similar (Fig 4A). Whereas, a significant decrease in the frequency of LAG-3^+^ occurred in silenced CD4^+^ T-cells from lung compared to untreated lung-derived T-cells at 24h (5-fold) and 48h (3-fold) post-infection (Fig 4B).

During this time, we also observed the presence of both IL-10 and IFN-γ in CD4^+^ T-cells in co-culture. The frequency of CD4^+^ T-cells expressing IFN-γ in both cultures increased over the 96h course of infection and this frequency was only slightly lower in the LAG-3 silenced CD4^+^ T-cells in comparison to the control co-cultures sans LAG-3 silencing (Fig 4C and 4D). In T-cells derived from blood of *Mtb*-infected NHPs, a slight decrease in frequency occurred at 48 and 72h post-infection (Fig 4C). In the co-culture model where CD4^+^ T-cells were isolated from lungs of *Mtb*-infected animals, the frequency of IFN-γ positive T-cells appeared to be lower at 24 and 48h post-infection in the LAG-3 silenced co-culture in comparison to the untreated culture (Fig 3D). Thus, a decrease in LAG-3 levels in the CD4^+^ T-cells of macaques due to RNA interference (RNAi) and the concomitant reduction in *Mtb* levels was not characterized by a corresponding increase in IFN-γ levels. Interestingly, a similar frequency of IL-10 positive CD4^+^ T-cells in LAG-3 silenced CD4^+^ T-cells was observed when compared to untreated cells (Fig 4E and 4F). In CD4^+^ T-cells isolated from blood, a decrease in the frequency of IL-10 positive T-cells was observed at 48 and 72h post-infection (Fig 4E). Similarly, a decrease in the frequency of IL-10 positive CD4^+^ T-cells occurred in lung-derived T-cells at 24 and 48h post-infection (Fig 4D).

### The effect of LAG-3 silencing on cytokine production during co-culture

**Fig 5:**
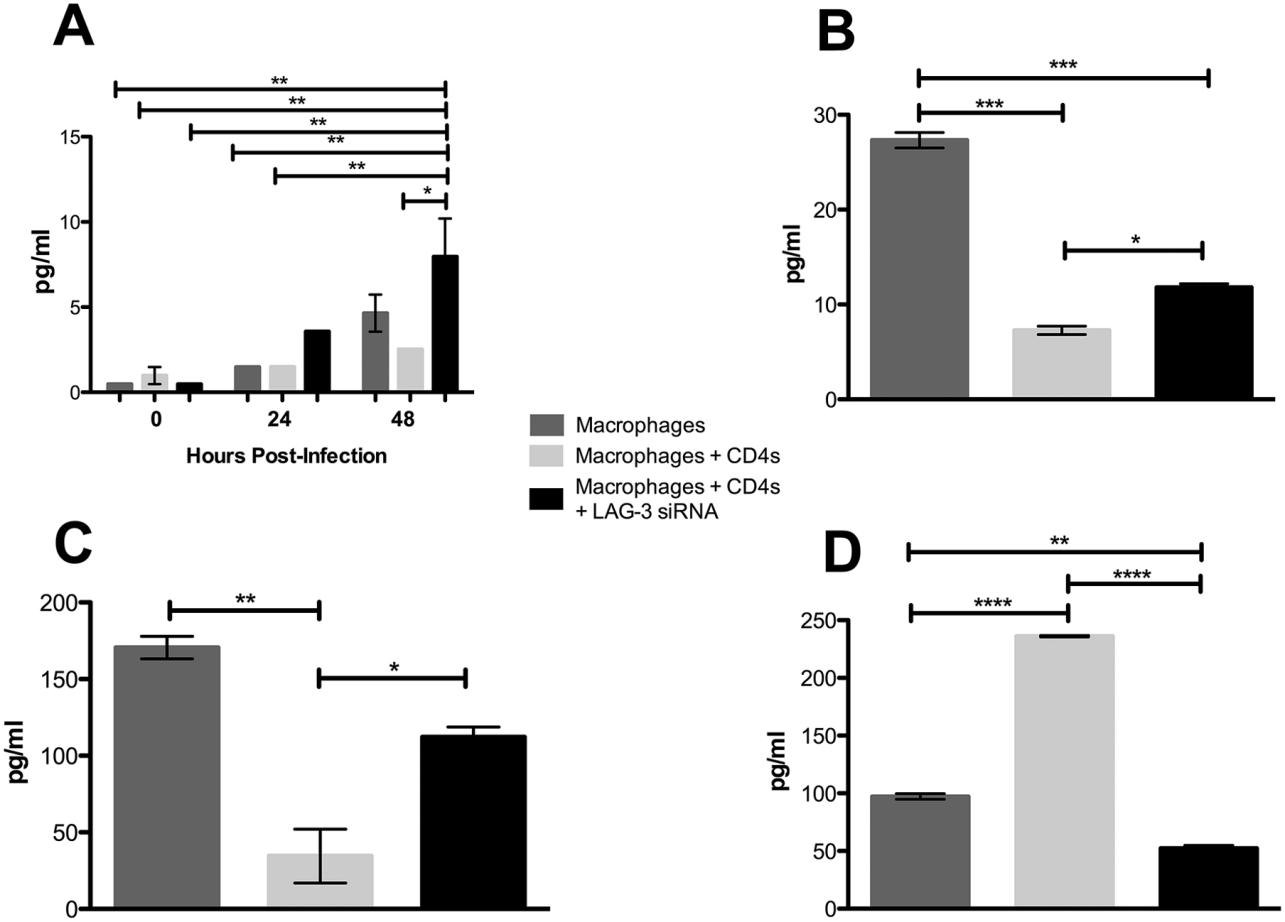
The effect of LAG-3 silencing on cytokine production in co-cultures supplemented with CD4^+^ T-cells from the blood of *Mtb*-infected rhesus macaques. (**A**) Production of IFN-γ in *Mtb*-infected macrophage culture, untreated co-cultures, and LAG-3 silenced co-cultures at 0, 24, and 48h post-infection. (**B**) The presence of IL-6 and (**C**) CXCL11 in *Mtb*-infected macrophage culture, untreated co-culture, and LAG-3 silenced co-culture at 48h post-infection. (**D**) Levels of MIF in *Mtb*-infected macrophage culture, untreated co-culture, and LAG-3 silenced co-culture supernatant at 24h post-infection. All samples were measured in pg/ml. Statistical significance was determined using a one-way ANOVA in Prism 6, using multiple comparisons to compare each mean, with the Tukey multiple comparison test; the mean and SEM are represented by horizontal bars. *p < 0.05, **p < 0.01, ***p < 0.001, ****p < 0.0001.

Sizeable differences in production of certain cytokines were observed in culture supernatants within the various groups. 48h post-infection, we detected a significant increase in the concentration of IFN-γ in the co-cultures with blood derived CD4^+^ T-cells where LAG-3 expression was silenced relative to those where silencing was not performed (Fig 5A). Here, a measurable increase in IFN-γ in the LAG-3 silenced co-culture and a decrease in the untreated co-culture was observed whereas the mean IFN-γ concentration in the infected macrophages alone was at an intermediate concentration. Silencing of LAG-3 also resulted in a significantly greater concentration of IL-6 (Fig 5B) and CXCL11 (Fig 5C) than controls. A contrasting pattern was observed for the inflammatory mediator macrophage migration inhibitory factor (MIF) [58]. The highest concentration of MIF was found to be in the untreated co-cultures, while the siRNA treated co-cultures had significantly decreased levels (Fig 5D).

**Fig 6:**
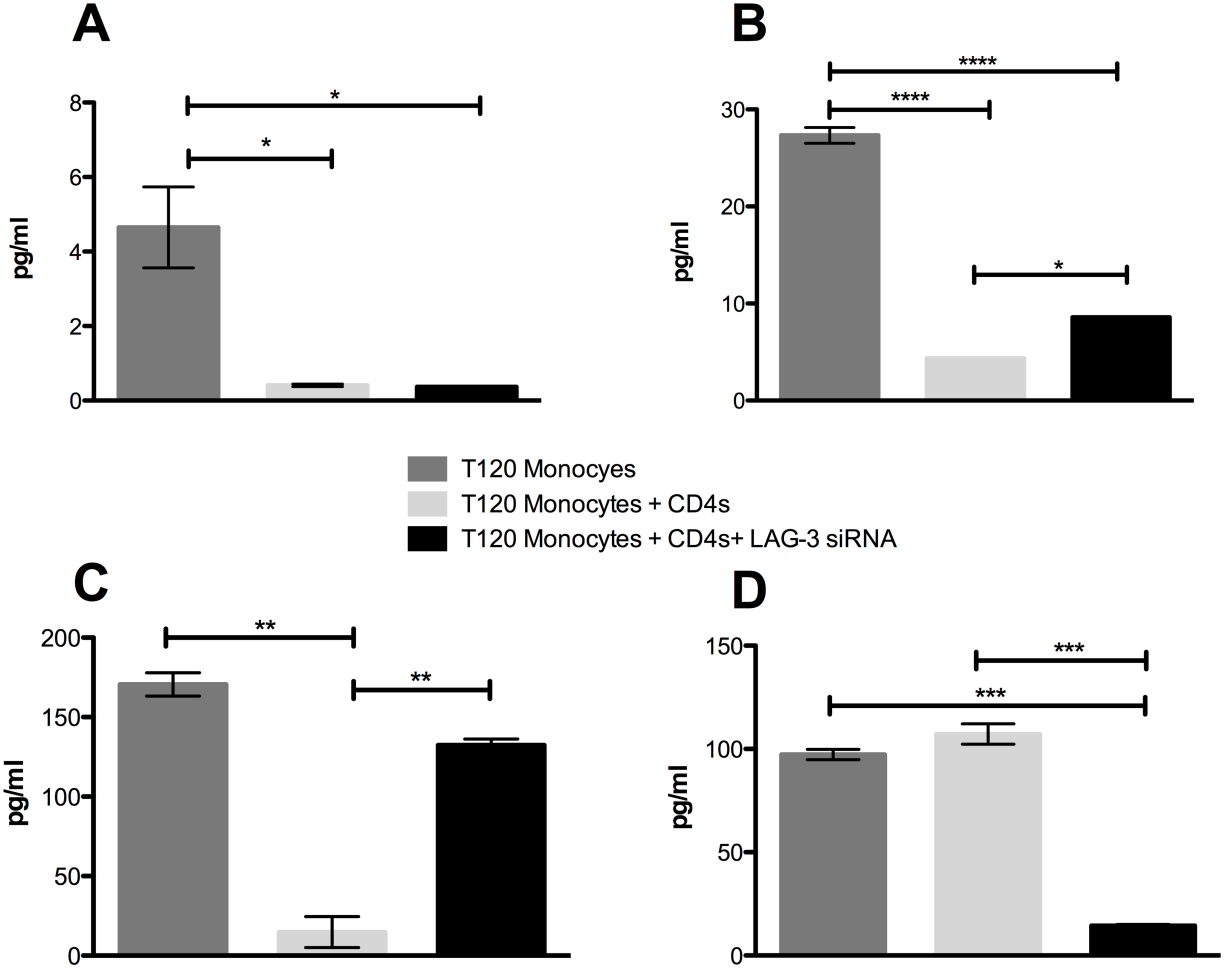
The effect of LAG-3 silencing on cytokine production in co-cultures supplemented with CD4^+^ T-cells isolated the lung of *Mtb*-infected rhesus macaques. (**A**) Concentrations of IFN-γ, (**B**) IL-6 and (**C**) CXCL11 in *Mtb*-infected macrophage culture, untreated co-culture, and LAG-3 silenced co-culture at 48h post-infection. (**D**) The presence of MIF in *Mtb*-infected macrophage culture, untreated co-culture, and LAG-3 silenced co-culture at 24h post-infection. All samples were measured in pg/ml. Statistical significance was determined using a one-way ANOVA in Prism 6, using multiple comparisons to compare each mean, with the Tukey multiple comparison test; the mean and SEM are represented by horizontal bars. *p < 0.05, **p < 0.01, ***p < 0.001, ****p < 0.0001.

Next, we went on to observe the differences between *Mtb*-infected macrophage cultures, and untreated and LAG-3 siRNA treated co-cultures, where the CD4^+^ T-cells had been isolated from lung of *Mtb*-infected NHPs. In these cultures, concentrations of IFN-γ were significantly lower in both co-culture subsets when compared to the macrophage alone culture (Fig 6A). It is possible that this may be due to these specific CD4^+^ T-cells being from an immunosuppressive environment within the lungs [59]. In *Mtb*-infected NHPs, the frequency of CD4^+^ T-cells expressing LAG-3 was earlier shown by us to be significantly greater within the lung when compared to blood from the same animals [32]. Similar results were observed with the lung-derived CD4^+^ T-cells for both pro-inflammatory proteins IL-6 and CXCL11 as was previously observed with the blood-derived co-cultures, with levels being significantly lower in the untreated co-culture group, and significantly greater in the LAG-3 silenced co-culture (Fig 6B and 6C). The same was the case with MIF, where the silencing of LAG-3 resulted in a significantly decreased concentration (Fig 6D). Thus, a similar pattern of cytokine production occurred in co-cultures in response to inhibition of LAG-3 signaling, regardless of the source of CD4^+^ T-cells. In general, the silencing of LAG-3 led to an increase in production of pro-inflammatory cytokines (e.g. IFN-γ, IL-6 and CXCL11), and a decrease in those known to be involved in immune inhibition (e.g. IL-10).

### Transcriptomic analysis of the LAG-3 depleted co-culture model

We next used a sequential transcriptomic approach to analyze the impact of LAG-3 silencing in blood- or lung-derived macaque CD4^+^ T-cells on the killing of *Mtb* within macaque macrophages. We first studied the impact of *Mtb* infection in macrophages at the 24h time-point, then the impact of adding lung-derived CD4^+^s to these cultures, and finally the impact of adding lung-derived CD4^+^ T-cells where LAG-3 expression had been silenced. ~250 genes exhibited significant perturbation in levels of expression in macrophages infected with *Mtb* for 48h, relative to uninfected cells, with ~150 exhibiting induction. The genes with strongest induction included chemokines (e.g. CXCL11, 84-fold; CXCL10, 18-fold; CXCL9, 9-fold; CCL2, 9-fold) and cytokines (e.g. IL6, 54-fold; IL12B, 10-fold; IL1B, 9-fold;) etc. (Table 1) consistent with the infection of host phagocytes with *Mtb* reported earlier by us [33, 34].

**Table 1:** Log_2_ average fold change values of gene expression for select immune function genes amongst biological replicate samples from *Mtb*-infected macrophages (24h) as well as the two co-culture sets (*Mtb*-infected macrophages (24h) + infected lung CD4^+^ T-cells; *Mtb*-infected macrophages (24h) + infected lung CD4^+^ T-cells where LAG-3 expression was silenced by RNAi).

Gene (Symbol)	Macs+*Mtb*	Macs+*Mtb*+ILU CD4	Macs+*Mtb*+ILU CD4 (LAG3 siRNA)
CCL2	1.53	0.15	-2.37
CCL3	1.24	0.97	4.29
CCL5	0.74	0.15	-2.37
CCL15	0.98	0.84	-2.06
CCL18	1.59	-0.79	-0.86
CCR5	0.61	-0.18	0.86
CCR7	1.08	-0.30	4.24
CXCL2	1.69	1.42	4.57
CXCL6	0	0	5.87
CXCL9	1.07	0.87	-3.94
CXCL13	2.25	3.25	3.10
CXCL14	2.05	0	1.12
CXCR6	1.20	0.97	1.33
GADD45A	2.90	0.68	3.18
IL1R2	1.34	1.36	0.42
IL1RL1LG	0.65	0.10	0.65
IL12RB2	0.97	0.86	2.22
IL17RC	0.60	-0.08	0.18
IL21R	0.92	0.65	2.15
IL23A	1.95	0.53	2.15
IL27RA	0.95	0.71	-0.80
IL4R	0.63	0.15	0.89
NFIL3	1.48	0.26	1.68
PTGS2	1.66	1.40	5.36

Significantly more genes exhibited perturbation in experiments where CD4^+^ T-cells obtained from the lungs of infected rhesus macaques were added to infected macrophages, with or without RNAi. This was unsurprising, both because CD4^+^ T-cells would be expected to bring their own transcriptomic signature, but also because their interaction with *Mtb*-infected macrophages could result in further changes to gene-expression. The expression of ~1300 genes was induced 24h after infected lung CD4^+^ T-cells were added to *Mtb*-infected macrophages, with the expression of ~1700 genes being repressed. The expression of a very similar number (~1400 genes with induced and ~1700 genes with repressed) was perturbed when lung-derived CD4^+^ T-cells had been silenced by specific RNAi, were added to *Mtb*-infected macrophages.

The genes with strongest induction in co-cultures included membrane-spanning 4-domains, subfamily A, member 6A (MS4A6A), transcript variant 3 (120-fold), alcohol dehydrogenase 7 (ADH7) (41-fold), transforming growth factor, beta-induced (TGFBI) (36-fold), CXCL11 (29-fold), CXCL10 (14-fold), CXCL9 (13-fold) and CCL2 (7-fold). However, the expression of most pro-inflammatory cytokines produced by infected macrophages (e.g. IL6, IL1B, IL12B etc.), was abrogated in the macrophage-T cell co-cultures (Table 1). Thus, the significant pro-inflammatory response triggered by the infection of macrophages by *Mtb* was curtailed upon addition of lung CD4^+^ T-cells. However, the expression of numerous immune function genes, particularly those belonging to the cytokine/chemokine families was induced to a higher level in co-cultures where LAG-3 expression was silenced (Table 1), correlating with greater bacterial killing and revealing a definite immunosuppressive function for LAG-3 in the context of TB. The genes with the largest level of induction included MS4A6A, transcript variant 3 (140-fold) and ADH7 (69-fold). Amongst chemokines, the expression of CXCL11 (45-fold), CXCL10 (18-fold), CXCL9 (15-fold) and CCL2 (5-fold) remained unperturbed relative to the second group, while the expression of pro-inflammatory cytokines was not detected.

**Fig 7:**
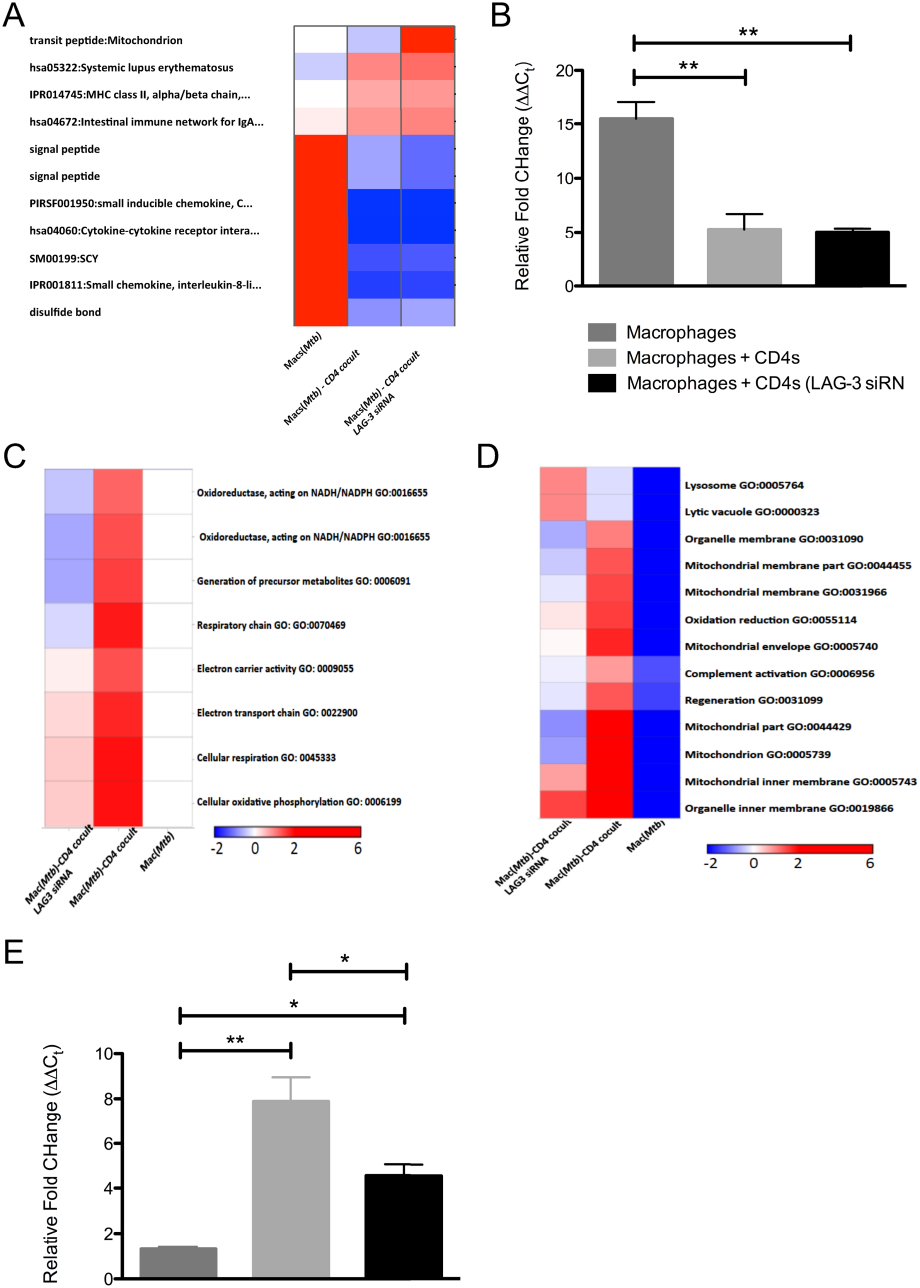
Global transcriptomic impact of silencing LAG-3 expression in macrophage-T-cell co-cultures during *Mtb* infection. Hierarchical clustering of specific gene ontology (GO) categories keywords or pathways revealed that immune activation/inflammation genes were represented to significantly higher levels in macrophages infected with *Mtb* and to lower levels in co-cultures (**A**). Mitochondrial electron transport related categories were significantly overrepresented in co-cultures of macrophages and lung derived CD4^+^ T-cells (**B** and **C**), but the over representation of these functions was ameliorated in co-cultures where LAG-3 expression was silenced by RNAi (**B** and **C**). The red color represents higher significance while the blue color represents lower significance. The values represented are negative log_10_ of significance p-values of accumulation using DAVID.

Due to the high similarly in transcriptomic results from two co-cultures, we used DAVID as a tool to understand minute differences therein. The two groups with CD4^+^ T-cells added to *Mtb*-infected macrophages appeared quite different from the *Mtb*-infected macrophage only group with the addition of T cells ameliorating a highly proliferative and inflammatory response in macrophages (Fig 7A). The expression of IL1B was maximally induced in infected macrophages (~40-fold) by RT-PCR, relative to the co-cultures (Fig 7B), validating the higher cytokine storm experienced by the pathogen in macrophages. Differences between the untreated and the LAG-3 specific siRNA groups, where CD4^+^ T-cells were co-cultured with infected macrophages, were centered on cellular respiration and electron transport chain. Thus, genes involved in these processes (e.g. GO terms cellular oxidative phosphorylation; cellular respiration; electron transport chain; electron carrier activity; respiratory chain; oxidoreductase, acting on NADH/NADPH etc.) were induced to significantly higher levels in co-cultures where LAG-3 expression was not silenced in CD4^+^ T-cells relative to where it was (Fig 7C). Furthermore, the enrichment of mitochondria-specific GO terms (e.g. organelle membrane/inner membrane; mitochondrial membrane/inner membrane; mitochondrion and mitochondrial envelope) in co-cultures of *Mtb*-infected macrophages with CD4^+^ T-cells was reduced when LAG-3 expression was silenced in T-cells prior to co-culturing (Fig 7D). On the other hand, genes belonging to GO terms lysosome and lytic vacuole were enriched in LAG-3 silenced co-cultures. The expression of SOD2 (superoxide dismutase 2, mitochondrial) was found to be highest in co-cultures of infected macrophages and CD4^+^ T-cells, followed by co-cultures where the T-cells has been incubated with LAG-3 specific siRNA, by RT-PCR, and these differences were statistically significant (Fig 7E). Since the *Mtb*-load difference between the two co-culture groups was not huge, especially when compared to the *Mtb*-infected macrophage cultures, it is unlikely that differences in mitochondrial gene-expression are a result of changes in inflammation. Our results therefore, strongly suggest that LAG-3 specific modulation of Th1 responses is mediated by interference with lysosomal function and enhanced mitochondrial electron transport. LAG-3 could therefore modulate adaptive immunity to *Mtb* infection by interfering with the mitochondrial apoptosis pathway.

## Discussion

*Mtb* owes is pathogenic success to its remarkably different strategy of interacting with its human host, with which it has co-evolved [4]. Thus, while most pathogens attempt to mask their immunogenicity, *Mtb* is rather unique in being able to elicit extremely high immune responses, as exemplified by the use of BCG as an adjuvant^4^. This is counterintuitive and *Mtb* appears to have evolved to retain and promote its immunogenicity. This is crucial in order to generate the classical tissue damage associated with TB—which is itself necessary for the transmission of *Mtb*. However, in order to complete this cycle, *Mtb* must first successfully persist in the wake of strong immune responses it elicits. Several lines of evidence indicate that *Mtb* successfully modulates host innate and adaptive immune responses [60]. Furthermore, IFN-γ is critical for the control of *Mtb*, which interferes with IFN-γ signaling at several steps [61, 62].

Recent studies show active subversion of specific granuloma immune mechanisms by *Mtb* within the granuloma. The expression of LAG-3 is highly induced in the granuloma during active TB [32] and occurs largely on CD4^+^ T-cells [32]. LAG-3 is a marker for chronically exhausted CD4 T cells [28] and its expression correlates with suppressed CD4^+^ T-cell responses [63]. Additionally, the expression of LAG-3 has been shown to facilitate the immunomodulatory function of Tregs [64] and NK/NKT cells [65] in a variety of scenarios including autoimmunity and cancer.

Blockading LAG-3 allowed CD4 cells to kill significantly more *Mtb*, accompanied by increased IFN-γ- and depressed IL-10-signaling. Together with our prior observation that LAG-3 is chiefly expressed on lung CD4^+^ T-cells that also co-express IL-10 [32], these results clearly show that IL-10-LAG-3 axis is crucial for the modulation of CD4^+^ T-cell responses to *Mtb* within the granuloma. Consistent with our previously published work, current data suggests that LAG-3 expression primarily occurs in tissues (in this case lung, and specifically the granuloma) rather than in the periphery [32]. Similar observations were made during the course of the current study. Silencing LAG-3 expression in blood- rather than lung-derived CD4s led to significant restriction of *Mtb*, likely due to an incomplete effect of RNAi on the levels of a stable protein in the cell membrane fraction (Fig 3). We conclude that *Mtb* actively reprograms the phenotype of antigen-specific T-cells at the site of infection for its own benefit. In support of this, only a fraction of total T-cells in the *Mtb*-induced lung lesion express high levels of protective cytokines [57].

To further elucidate the mechanism of LAG-3 we hope to take advantage of mice with LAG-3 knockouts and the possibility of targeted removal of this allele in murine T-cells. It may also be possible to perform *in-vivo* inhibition of LAG-3 signaling in macaques to better understand its role, using fusion proteins or depleting antibodies. Inhibition of LAG-3 activity could potentially be a host-based target towards, along the lines of immunotherapies against cancer that are being developed using LAG-3.

## Supporting information

S1 Fig Experimental timelines. (A) timeline illustrating the experimental design to observe the difference in the initial uptake of *Mtb*, the bacterial burden over the course 96 hrs post-infection, and the cellular morphology between T24 monocytes and T120 differentiated macrophages. (B) the timeline of the co-culture experiment designed to observe the effect of LAG-3 silencing in T-cells over the course of *Mtb* infection.
(PDF)

S2 Fig LAG-3 mRNA sequence in rhesus macaque (*Macaca mulatta*) genome and LAG-3 siRNA targets. (A) This diagram shows the entire mRNA sequence of LAG-3 specific for rhesus macaques, as well as sense and antisense sequence of the LAG-3 siRNA molecules. The yellow highlighted areas in the mRNA sequence illustrate the regions targeted by the siRNAs. Additionally, the green and the red highlighted areas indicate the start and end sequences, respectively. The letters in red denote the area between the exons. (B) The expression levels of LAG-3 in T-cells that were treated with specific siRNA (25 nM), or treated with NTC with the transfection reagent at 24 post-siRNA transfection. RT-PCR was utilized to measure the fold change of LAG-3 mRNA expression by calculating 2^-ΔΔCt^, normalized to β-actin and control T-cell values. Red bars indicate untreated T-cells and green bars show T-cells treated with LAG-3 siRNA.
(PDF)

S3 Fig The gating strategies utilized in Fig 4. (A) Gating strategy used to select for CD4^+^ T-cells in all images. Here, we first gated for the lymphocyte population, then selected singlets, and then went on to select for CD3^+^ T cells before graphing for CD4^+^ vs. CD8^+^ in order to obtain CD4^+^CD8^+^ T-cells. Gating strategy used to select for CD4^+^ T-cells expressing LAG-3 (B), IL-10 (C) and IFN-γ (D). A quadrant gate was set for IL-10 vs. IFN-γ and we analyzed all IFN-γ^+^ cells.
(PDF)

S1 Table This document provides details of flow cytometry (A) and confocal microscopy (B) in terms of the different antibodies used, their dilutions etc. (PDF)

## Abbreviations

ADC: albumin-dextrose-catalase
ADH7: alcohol dehydrogenase 7
DAVID: The Database for Annotation, Visualization and Integrated Discovery
GO: Gene Ontology
LAG-3: lymphocyte activation gene-3
MIF: macrophage migration inhibitory factor
MOI: multiplicity of infection
MS4A6A: membrane-spanning 4-domains, subfamily A, member 6A
*Mtb*: *Mycobacterium tuberculosis*
NHP: non-human primate
NTC: no template control
RNAi: RNA interference
siRNA: small interfering RNA
TB: tuberculosis
TGFBI: transforming growth factor, beta-induced
Treg: regulatory T cell
TST: tuberculin skin test
