# Supplementary material for: LAG-3 potentiates the survival of Mycobacterium tuberculosis in host phagocytes by modulating mitochondrial signaling in an in-vitro granuloma model
Source: PLoS One. 2017 Sep 7;12(9):e0180413. doi: 10.1371/journal.pone.0180413 (PMC5589099; doi:10.1371/journal.pone.0180413)
Supplement: S1 Fig — (A) timeline illustrating the experimental design to observe the difference in the initial uptake of Mtb, the bacterial burden over the course 96 hrs post-infection, and the cellular morphology between T24 monocytes and T120 differentiated macrophages. (B) the timeline of the co-culture experiment designed to observe the effect of LAG-3 silencing in T-cells over the course of Mtb infection. (PDF) [file pone.0180413.s001.pdf]

Supplemental Figure 1

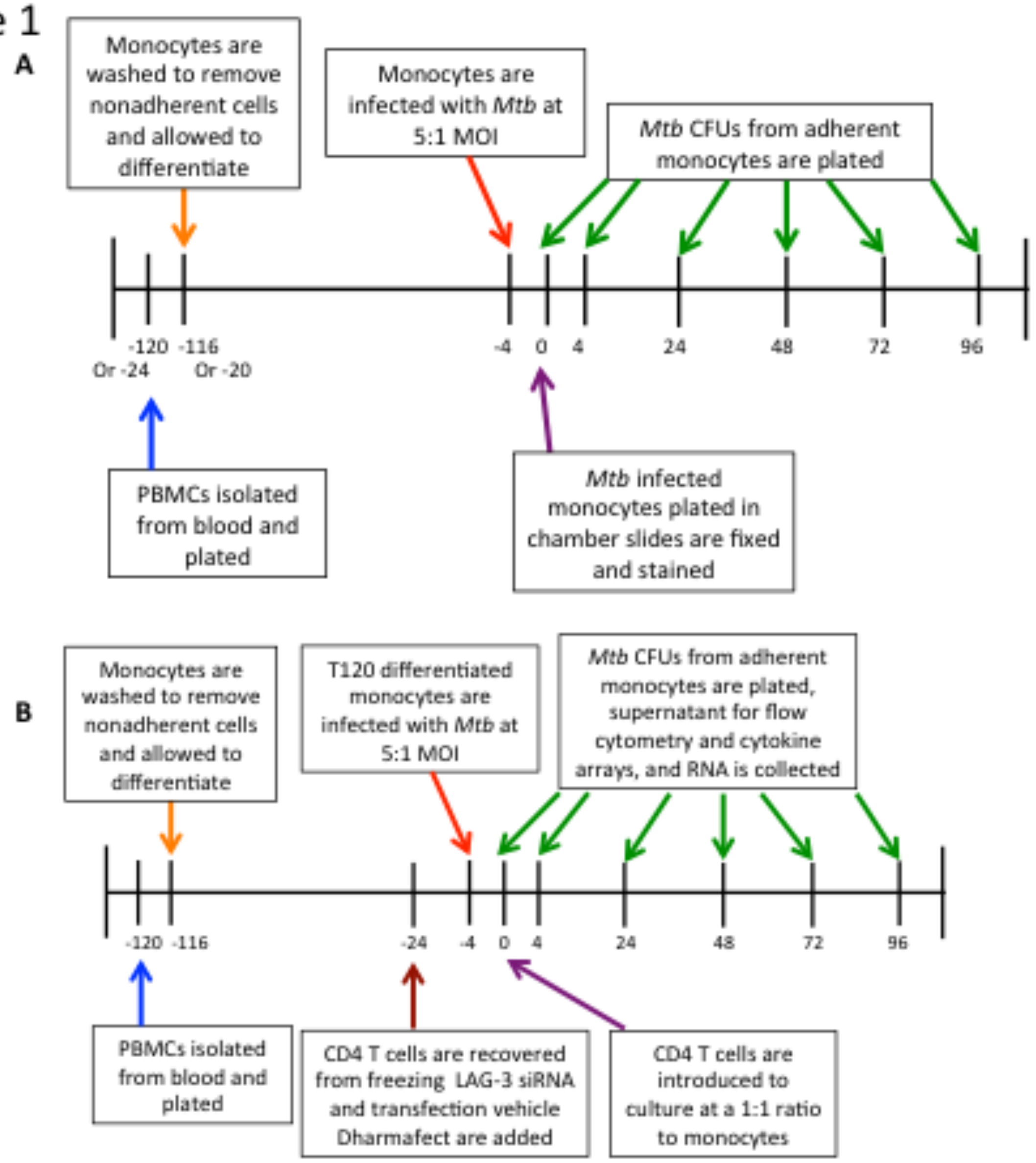

SUPPLEMENTAL FIGURE 1 Experimental Timelines (**A**) Timeline illustrating the experimental design to observe the differences in initial uptake of *Mtb*, the bacterial burden over the course of 96 hours post-infection, and the cellular morphology between T24 monocytes and T120 differentiated macrophages. (**B**) The timeline of the co-culture experiment designed to observe the effect of LAG-3 silencing in T cells over the course of *Mtb* infection the above-described model.
