# Supplementary material for: LAG-3 potentiates the survival of Mycobacterium tuberculosis in host phagocytes by modulating mitochondrial signaling in an in-vitro granuloma model
Source: PLoS One. 2017 Sep 7;12(9):e0180413. doi: 10.1371/journal.pone.0180413 (PMC5589099; doi:10.1371/journal.pone.0180413)
Supplement: S3 Fig — (A) Gating strategy used to select for CD4+ T-cells in all images. Here, we first gated for the lymphocyte population, then selected singlets, and then went on to select for CD3+ T cells before graphing for CD4+ vs. CD8+ in order to obtain CD4+CD8+ T-cells. Gating strategy used to select for CD4+ T-cells expressing LAG-3 (B), IL-10 (C) and IFN-γ (D). A quadrant gate was set for IL-10 vs. IFN-γ and we analyzed all IFN-γ+ cells. (PDF) [file pone.0180413.s003.pdf]

Supplemental Figure 3

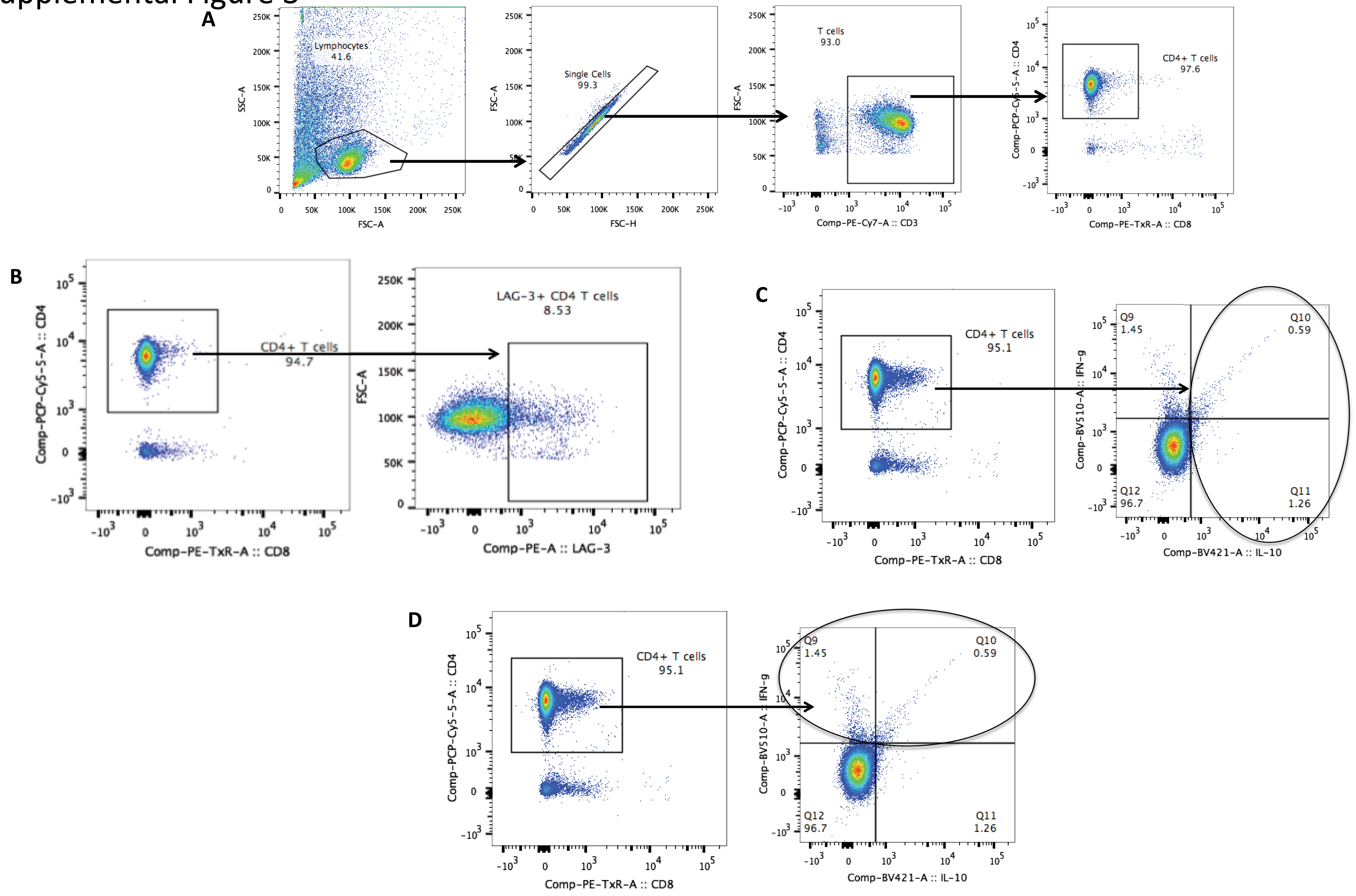

SUPPLEMENTAL FIGURE 3 The gating strategies utilized in **Fig. 3**. (**A**) The gating strategy used to select for CD4 T cells in all images. Here, we first gated for the lymphocyte population, before gating for singlets, we then went on to select for $CD3^+$ T cells before graphing for CD4 vs. CD8 in order to obtain $CD4^+CD8^-$ T cells. (**B**) The gating strategy used select for CD4 T cells expressing LAG-3. (**C**) The gating strategy used select for CD4 T cells expressing IL-10. A quadrant gate had been set for IL-10 vs. IFN-γ, and we analyzed all $IL\text{-}10^+$ cells. (**D**) The gating strategy used select for CD4 T cells expressing IFN-γ. A quadrant gate had been set for IL-10 vs. IFN-γ, and we analyzed all $IFN\text{-}\gamma^+$ cells.
