# Supplementary material for: LAG-3 potentiates the survival of Mycobacterium tuberculosis in host phagocytes by modulating mitochondrial signaling in an in-vitro granuloma model
Source: PLoS One. 2017 Sep 7;12(9):e0180413. doi: 10.1371/journal.pone.0180413 (PMC5589099; doi:10.1371/journal.pone.0180413)
Supplement: S2 Fig — (A) This diagram shows the entire mRNA sequence of LAG-3 specific for rhesus macaques, as well as sense and antisense sequence of the LAG-3 siRNA molecules. The yellow highlighted areas in the mRNA sequence illustrate the regions targeted by the siRNAs. Additionally, the green and the red highlighted areas indicate the start and end sequences, respectively. The letters in red denote the area between the exons. (B) The expression levels of LAG-3 in T-cells that were treated with specific siRNA (25 nM), or treated with NTC with the transfection reagent at 24 post-siRNA transfection. RT-PCR was utilized to measure the fold change of LAG-3 mRNA expression by calculating 2-ΔΔCt, normalized to β-actin and control T-cell values. Red bars indicate untreated T-cells and green bars show T-cells treated with LAG-3 siRNA. (PDF) [file pone.0180413.s002.pdf]

Supplemental Figure 2

A

Macaque LAG-3

NCBI Reference Sequence: XM_001108923.2

```
AGCTTTCCTCTGGATTCTGGCCTCTGGTCATCCCTCCCCACCCTCTCTCCAAGGCTCTCTCCTGGTCTCC
CTTCTTCTAGATCCTCTTCCTCCACCTCCCTATCTGCAGAACTTCTCCTTTGCCCCCCCACCCCCTACCA
CTGCCCACTTTCCTTTTCTGACCTCCTTTTGGAGGGCTCAGTGCTGCCCAGACCATAGGAGAGATGTGGG
AGGCTCAGTTCCTGGGCTTGCTGTTTCTGCAGCCGCTCTGGGTGGCTCCAGTGAAGCCTCCCCAGCCAGG
GGCTGAGATCTCGGTGGTGTGGGCCCAGGAGGGGGCTCCTGCCCAGCTCCCCTGCAGCCCCACAATCCCC
CTCCAGGATCTCAGCCTTCTGCGAAGAGCAGGGGTCACTTGGCAGCATCAACCAGACAGTGGCCCGCCCG
CTCCCGCCCCCGGCCACCCCCCGGCCCCCGGCCATCGCCCGGCGGCGCCCTACTCTTGGGGGCCCAGGCC
CCGCCGCTACACGGTGCTGAGCGTGGGTCCTGGAGGCCTGCGCAGCGGGAGGCTGCCCCTGCAGCCCCGC
GTCCAGCTGGATGAGCGCGGCCGGCAGCGCGGGGACTTCTCGCTGTGGCTGCGCCCAGCCCGGCGCGCGG
ACGCCGGCGAGTACCGCGCCACGGTGCACCTCAGGGACCGCGCCCTCTCCTGCCGCCTTCGTCTGCGCGT
GGGCCAGGCCTCGATGACTGCCAGCCCCCCAGGGTCTCTCAGGACCTCTGACTGGGTCATTTTGAACTGC
TCCTTCAGCCGCCCTGACCGCCCAGCCTCTGTGCATTGGTTCCGGAGCCGTGGCCAGGGCCGAGTCCCTG
TCCAGGGGTCCCCCCATCACCACTTAGCGGAAAGCTTCCTCTTCCTGCCCCATGTCGGCCCCATGGACTC
TGGGCTCTGGGGCTGCATCCTCACCTACAGAGATGGCTTCAATGTCTCCATCATGTATAACCTCACTGTT
CTGGGTCTGGAGCCCGCAACTCCCTTGACAGTGTACGCTGGAGCAGGTTCCAGGGTGGAGCTGCCCTGCC
GCCTGCCTCCTGCTGTGGGGACCCAGTCTTTCCTTACTGCCAAGTGGGCTCCTCCTGGGGGAGGCCCTGA
CCTCCTGGTGGCTGGAGACAATGGCGACTTTACCCTTCGACTAGAGGATGTAAGCCAGGCCCAGGCTGGG
ACCTACATCTGCCATATCCGTCTACAGGGACAGCAGCTCAATGCCACTGTCACATTGGCAATCATCACAG
TGACTCCCAAATCCTTTGGGTCACCTGGCTCCCTGGGGAAGCTGCTTTGTGAGGTGACTCCAGCATCTGG
ACAAGAACACTTTGTGTGGAGCCCCCTGAACACCCCATCCCAGAGGAGTTTCTCAGGACCATGGCTGGAG
GCCCAGGAAGCCCAGCTCCTTTCCCAGCCTTGGCAATGCCAGCTGCACCAGGGGGAGACGCTTCTTGGAG
CAGCAGTATACTTCACAGAACTGTCTAGCCCAGGTGCACAACGCTCTGGGAGAGCCCCAGGGGCCCTCCG
AGCAGGCCACCTCCCGCTGTTTCTCATCCTTGGTGTCCTTTTTCTGCTCCTTTTGGTGACTGGAGCCTTT
GGCTTTCACCTTTGGAGAAGACAGTGGCGACCAAGAAGATTTTCTGCCTTAGAGCAAGGGATTCACCCTC
CGCAGGCTCAGAGCAAGATAGAGGAGCTCGAGCAAGAACCGGAGCTGGAACCAGAGCCGGAGCTGGAGCG
CGAGCTGGGGCCGGAGCCCGAGCCGGGGCCTGAGCCCGAGCCGGAGCAGCTCTGA
```

```
LAG-3 siRNA CCUGGUGGCUGGAGACAAU        Sense
LAG-3 siRNA AUUGUCUCCAGCCACCAGGUU      Antisense
```

B

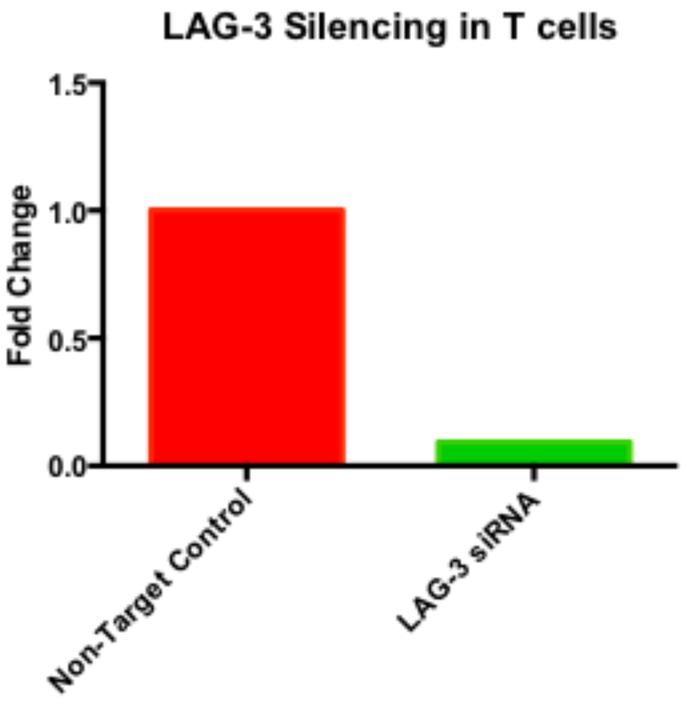

SUPPLEMENTAL FIGURE 2 LAG-3 mRNA sequence in rhesus macaque and LAG-3 siRNA targets. (**A**) This diagram shows the entire mRNA sequence of LAG-3 specific for rhesus macaque, as well as the sense and antisense sequence of the LAG-3 siRNA. The yellow highlighted areas in the LAG-3 mRNA sequence illustrate the regions that the siRNAs are targeting. Additionally, the green and red highlighted areas indicate the start and end sequences, respectively. The letters in red denote areas between exons. (**B**) The expression levels of LAG-3 in T cells that were treated with LAG-3 specific siRNA (25nM concentration), or treated with the NTC with transfection reagent at 24 hours post-transfection of the siRNAs. RT-PCR was utilized to measure the fold change of LAG-3 mRNA expression through the calculation of the $2^{-\Delta\Delta Ct}$, where normalization was calculated with β-actin and the control T cells. Red bars indicate control T cells and green bars show T cells treated with LAG-3 siRNA.
