# Supplementary material for: LAG-3 potentiates the survival of Mycobacterium tuberculosis in host phagocytes by modulating mitochondrial signaling in an in-vitro granuloma model
Source: PLoS One. 2017 Sep 7;12(9):e0180413. doi: 10.1371/journal.pone.0180413 (PMC5589099; doi:10.1371/journal.pone.0180413)
Supplement: S1 Table — (PDF) [file pone.0180413.s004.pdf]

Supplemental Table 1.

A. Details of flow cytometry

The samples were stained with the following fluorochrome-conjugated antibodies in stain buffer (PBS, 1% BSA, 0.5% sodium azide):

CD4	PerCP-Cy5.5, BD Biosciences)
IL-10	BV421, Biolegend, San Diego, CA)
IFN-γ	BV510, BD Biosciences)
LAG-3	PE, R&D Systems, Minneapolis, MN)
CD8	PE-TxR, Invitrogen)
CD3	PE-Cy7, BD Biosciences)

Samples were run on a BD Fortessa flow cytometer (BD Biosciences). Flow cytometric analysis was performed with FlowJo software (version 10; Treestar, Ashland, OR).

B. Details of confocal microscopy

Primary antibodies against the following proteins were used:

MAC387	(1:50, IgG1 mouse, Dako)
Mtb	(1:100, rabbit, Molecular Probes)
TO-PRO 3	(1:2000, Molecular Probes)

The following secondary antibodies conjugated to fluorochromes from Molecular Probes at a 1:1000 concentration derived from goat:
Alexa Fluor 488 anti-mouse IgG1
Alexa Fluor 568 anti-rabbit.

Imaging was performed with a Leica True Confocal Laser Scanning Microscope SP2 laser scanning confocal microscope (Leica; Buffalo Grove, IL), and the images were analyzed with Velocity 3D image analysis software (Perkin Elmore; Waltham, MA)
